# Impact of Radiation on Dysphagia-Related Structures: A Dosimetric and Clinical Comparative Analysis of Three-Dimensional Conformal Radiotherapy (3D-CRT) and Intensity-Modulated Radiation Therapy (IMRT) Techniques in Patients With Head and Neck Cancer

**DOI:** 10.7759/cureus.58276

**Published:** 2024-04-14

**Authors:** Surendra Manam, Ravi Teja, Anand Rao PB, SK Azharuddin

**Affiliations:** 1 Radiation Oncology, GSL Medical College and General Hospital, Rajahmundry, IND; 2 Medical Oncology, GSL Medical College and General Hospital, Rajahmundry, IND

**Keywords:** dysphagia aspiration-related structures, intensity-modulated radiation therapy (imrt), head and neck squamous cell carcinoma (hnscc), head and neck cancer (hnc), head and neck oncology, dars

## Abstract

Introduction

Head and neck squamous cell carcinoma (HNSCC) is a significant health concern in India, with around one million new cases annually. The prevalence of HNSCC is notably high in Asia, especially in India, due to habits like tobacco chewing, betel nut usage, and alcohol consumption. Treatment typically involves a combination of surgery, radiation, chemotherapy, and biological therapy, aiming for tumor control while preserving function and quality of life. However, survivors often face long-term side effects like difficulty swallowing, leading to complications such as aspiration pneumonia. Intensity-modulated radiotherapy (IMRT) has shown promise in improving outcomes by sparing critical swallowing structures. Efforts to minimize radiation-related dysphagia are crucial for enhancing patients' quality of life post-treatment. Our study focuses on examining dosimetric parameters associated with dysphagia aspiration, alongside evaluating dysphagia grades in both treatment groups using the RTOG scale.

Material and methods

Patients with histologically confirmed non-metastatic head and neck carcinomas were included in our study in November 2018-April 2020. A total of 56 patients were taken into our study with 28 in each arm. They underwent radical radiotherapy (RT) with a total dose of 66-70 Gy, with or without concurrent chemotherapy, meeting specific inclusion criteria and excluding those receiving reirradiation or with distant metastasis. Patients were divided into two groups: Group I received three-dimensional conformal radiotherapy (3D-CRT), and Group II received IMRT. Treatment planning involved immobilization, CT imaging, delineation of target volumes and organs at risk, and contouring of swallowing structures. Dose-volume histogram parameters (mean dose, maximum dose, V30, V70, V80, D50, and D80) were used to assess mean dose to swallowing structures outside the planning target volume (PTV), with a mean dose constraint of 50 Gy. Dysphagia was evaluated using the RTOG criteria at baseline, during treatment, and six months post-treatment. Statistical analysis was performed using SPSS, with significance set at p < 0.05.

Results

In our study, the mean age at presentation differed slightly between the IMRT and 3D-CRT arms: 58 years versus 55 years, respectively. A higher proportion of patients in both arms experienced symptoms for three to six months, with 53.6% in 3D-CRT and 42.9% in IMRT. Stage distribution varied, with IV being most common in 3D-CRT and stage II in IMRT. Approximately 56% of patients in both groups had a history of smoking. Significant differences were observed in spinal cord dose between 3DCRT and IMRT techniques (p < 0.001). Similarly, a significant difference was found in the mean dose received by dysphagia aspiration-related structures (DARSs) between the 3D-CRT and IMRT arms (p = 0.04). Patients in the IMRT arm exhibited superior dysphagia grades compared to those in the 3D-CRT arm, with statistical significance observed in the third month (p = 0.008) and sixth month (p = 0.048).

Conclusion

Our study found a notable decrease in the mean DARS dose and reduced dysphagia severity at three and six months in the IMRT group compared to the 3D-CRT group. However, due to the diverse study population, establishing a definitive correlation between the DARS dose and dysphagia severity was challenging. Future large-scale studies are needed to validate these findings for improved preservation of DARS structures.

## Introduction

Cancer is a leading health problem in India, with over one million new cases annually, including more than 600,000 cases of head and neck squamous cell carcinoma (HNSCC) worldwide, with 60% being locally advanced [1]. India bears a significant burden of head and neck cancers, accounting for 30% of all cancer cases. The high incidence in India is primarily attributed to habits like chewing tobacco and betel nut, alcohol, and smoking. Treatment typically involves a multidisciplinary approach combining surgery, radiation, chemotherapy, and biological therapy to control tumors while preserving function and quality of life.

For patients with unresectable HNSCC, chemoradiation or radiation alone is often the preferred treatment for organ preservation. However, many survivors experience long-term toxicities, such as xerostomia and dysphagia, impacting their quality of life. Dysphagia following radiation therapy is a significant issue, with nearly 50% of patients reporting it as distressing [2]. Radiation-related damage to critical swallowing structures can lead to aspiration and pneumonia, often underreported in clinical trials, resulting in dietary changes, nutritional deficiencies, and prolonged feeding tube dependence [3]. Late radiation-associated dysphagia, characterized by delayed onset swallowing dysfunction, further exacerbates complications.

The introduction of intensity-modulated radiotherapy (IMRT) has shown promise in improving quality of life by preserving salivary function and reducing radiation doses to critical swallowing structures [4]. Studies emphasize the importance of sparing these structures to minimize dysphagia and its consequences, with the mean dose to the pharyngeal constrictor muscle being a strong predictor of swallowing impairment [5].

The present study aims to investigate the impact of radiation on dysphagia-related structures and compare dosimetric and clinical outcomes between three-dimensional conformal radiotherapy (3D-CRT) and IMRT techniques in patients with head and neck cancer.

## Materials and methods

All patients who attended the Department of Radiation Oncology at GSL Medical College and General Hospital, Rajahmundry, India, with histologically proven head and neck carcinomas, meeting the inclusion and exclusion criteria, were included in the study during the study period from November 2018 to April 2020 (18 months). A total of 56 patients were taken into our study with 28 in each arm. All the patients were subjected to baseline investigations, including blood parameters, chest X-ray, ultrasound abdomen, and MRI/CT scan of the local site. The study subjects were selected using systematic random sampling.

Patient selection criteria

The inclusion criteria include histopathologically confirmed non-metastatic carcinomas of the head and neck receiving radical radiotherapy (RT) with or without concurrent chemotherapy, Karnofsky performance status with at least 70% score.

The exclusion criteria include patients receiving reirradiation and distant metastasis.

Study design

Eligible patients included in the study were sequentially assigned to one of the arms: Group I included patients who were treated with the 3D-CRT technique. Group II included patients who were treated with the IMRT technique.

After proper evaluation and staging, the patients were admitted and sent for simulation. All the patients were treated with the Elekta Versa HD linear accelerator (Elekta, Stockholm) with 6 MV photons.

Radiotherapy technique and planning

After obtaining informed consent, they were immobilized from head to shoulders with a five-point thermoplastic cast in the supine position using an appropriate headrest. Planning CT images with a 3 mm slice thickness, enhanced with contrast, were obtained from the supraorbital ridge to the carina. Following the simulation CT scan, these images were transferred in a DICOM (Digital Imaging and Communications in Medicine) format to the Treatment Planning System (Monaco Version 6.00.01, Elekta). The baseline pre-treatment dysphagia score was recorded based on the questionnaires of the Radiation Therapy Oncology Group (RTOG) criteria.

Gross tumor volume (GTV) was delineated, considering both clinical and radiological extent. The clinical target volume (CTV) was delineated following RTOG guidelines, and subsequently, the planning target volume (PTV) was defined in accordance with established institutional protocols. The adjacent organs at risk that were contoured included the parotid gland, submandibular gland, spinal cord, brain stem, eyes, optic nerve, optic chiasma, lens, and mandible. To outline the swallowing apparatus, we defined the superior constrictor muscle from the caudal tips of the pterygoid plates to the upper edge of the hyoid bone, the middle constrictor muscle from the upper to the lower edge of the hyoid, and the inferior constrictor muscle from below the hyoid to the inferior edge of the cricoid cartilage. The larynx was delineated from the tip of the epiglottis to the bottom of the cricoid, while the esophagus was contoured from the lower border of the cricoid downward, corresponding to the caudal-most extent of the low neck target volumes, as suggested by Christianen et al. [6]. Contours were delineated for the segment of the dysphagia aspiration-related structure (DARS) located outside the PTV, with a mean dose constraint of 50 Gy implemented. Dose-volume histograms (DVHs) were used to evaluate the mean dose to midline swallowing (DARSs). Toxicity levels were determined based on RTOG radiation morbidity scoring criteria. Dysphagia was evaluated at the outset, weekly throughout radiation therapy, and again six months post-treatment completion. The radiation dose administered to critical swallowing structures was analyzed for its correlation with the severity of dysphagia.

Statistical analysis

All data were entered into an MS Excel sheet (Microsoft Corporation, USA), and statistical analysis was conducted using IBM SPSS Statistics for Windows, version 20.0 (released 2011, IBM Corp., Armonk, NY). Descriptive values are reported as mean ± SD (standard deviation) and percentages. The Student's t-test was employed to compare various categorical variables, while the chi-square test was used to assess the association between different categorical variables. A significance level of p < 0.05 was considered statistically significant for all analyses.

## Results

In our study, the mean age at presentation was 58 years in the IMRT arm and 55 years in the 3D-CRT. A higher proportion of patients in the 3D-CRT arm (53.6%) and in the IMRT arm (42.9%) presented with symptom (which include but not limited to dysphagia, odynophagia, and oral reactions) durations of three to six months. The most common stage of presentation in the 3D-CRT arm was IVA, whereas in the IMRT arm, it was stage II. The hypopharynx (35.7%) was the most common site of the primary tumor followed by the oral cavity (32.1%) in our study group. Approximately 56% of patients in both arms had a history of smoking in one form or another. There was a statistically significant difference in the mean dose received by DARSs between the 3D-CRT and IMRT arms (p-value = 0.04) (Table 1).

**Table 1:** Dosimetric parameters of the combined DARSs DARS: dysphagia aspiration-related structure; 3D-CRT: three-dimensional conformal radiotherapy; IMRT: intensity-modulated radiotherapy; V%: volume in percentage; D(Gy): dose in gray; V30Gy: percentage of volume receiving 30 Gy; D50%: dose received by 50% of the volume. All values in mean ± SD.

Combined DARSs	Group I: 3D-CRT	Group II: IMRT	P-value
Mean	53.89 ± 7.25	48.50 ± 12.31	0.04
V30	89.64 ± 10.28	73.33 ± 14.19	0.4
V50	69.24 ± 11.42	61.74 ± 11.07	0.33
V70	0.07 ± 0.13	3.60 ± 14.11	0.19
V80	0.00 ± 0.00	0.00 ± 0.00	-
D50	52.89 ± 8.57	50.03 ± 16.30	0.41
D80	39.76 ± 11.76	38.07 ± 18.84	0.68

In our study, three patients had grade 3 dysphagia in the IMRT arm (10%) and four patients in the 3D-CRT arm (14.2%) at six weeks post-radiation. Furthermore, we noted that the patients in the IMRT arm had better grades of dysphagia compared to those in the 3D-CRT arm, with statistical significance observed in the third month (p-value = 0.008) and the sixth month (p-value = 0.048).

## Discussion

Our study aimed to evaluate the degree of dysphagia following DARS-sparing IMRT during the course of radiation and six months following the completion of radiation using the RTOG questionnaire. We exclusively calculated the dose to the DARS located outside the PTV, without endeavoring to spare the DARS in close proximity or within the PTV volume. Our approach aligns with the methodology employed by Eishbruch et al., who utilized IMRT to mitigate doses of the DARS while maintaining target doses [7]. In our study cohort, the hypopharynx (35.7%) emerged as the predominant site of primary tumor, succeeded by the oral cavity (32.1%). By contrast, the research conducted by Caglar et al. and Eishbruch et al. exclusively focused on laryngeal and oropharyngeal tumors for their analyses, whereas Peponi and colleagues included all primary sites similar to that of our study [7,8,9].

Advancements in HNSCC treatments have resulted in improved response and locoregional control rates. However, despite these advancements, mortality rates remain a significant concern. Intensified treatments, such as chemo-radiotherapy (CRT) or altered fractionation RT have shown improved outcomes but are associated with severe early and late mucosal and pharyngeal toxicities. Oropharyngeal dysphagia, often underestimated, is a common symptom in HNC patients, attributed to various factors, including neurological, structural, and iatrogenic causes. It is crucial not to overlook dysphagia, as it can significantly impact the quality of life. In our study, we observed dysphagia as a challenging side effect, alongside mucositis.

Rates of acute and late swallowing dysfunction ranging from 15% to 63% and 3% to 21%, respectively, have been reported [10]. Eisbruch et al. proposed DARS-sparing IMRT as an advancement over standard IMRT, allowing for tailored dose delivery to the DARS while maintaining target volume doses, leading to improved response in dysphagia and quality of life. Patients predominantly cited difficulty with consuming solids as their primary swallowing issue. While xerostomia is a contributing factor, there is a significant correlation between the radiation dose and dysphagia severity [7].

In our present study, a 50 Gy constraint was applied to the DARS, yet meeting this dose constraint was not mandatory. This approach was influenced by Eishbruch et al., who also opted for a 50 Gy constraint for the DARS based on observations of the minimal dose required for most constrictors involved in stricture formation [7]. Similarly, Galloway et al. utilized a mean dose constraint of 50 Gy for DARS-sparing IMRT in laryngeal irradiation. However, in their study, achieving the 50 Gy constraint was not obligatory, and compromises to constrictor dose were necessary in cases where the tumor was in close proximity to the pharynx [11]. In a study conducted by Feng et al., they explored the application of IMRT to alleviate dysphagia and noted that maintaining the dose of the DARS below 45 Gy resulted in no instances of aspiration events [12]. Van der Molen et al. demonstrated that a mean dose exceeding 63 Gy to the superior constrictor muscles (SCM) and middle constrictor muscles (MCM) correlated with decreased swallowing quality [13]. Similarly, Feng et al. found that the pharyngeal constrictors collectively exhibited diminished swallowing quality under similar dose conditions [12]. In our study, the mean dose for the SCM and MCM in 3D-CRT was 57.55 Gy and 62.40 Gy, respectively, whereas in IMRT, it was 51.06 Gy and 59.37 Gy, respectively.

In our study, three patients had grade 3 dysphagia (10%) in the IMRT arm and four patients in the 3D-CRT arm at six weeks after radiation therapy. This differs from a study conducted by Forastiere et al., where 23% of radiation-treated patients were only able to consume liquid food [14]. In another study by Caglar et al., 32% of patients experienced clinically significant aspiration, while 37% developed strictures. Peponi et al., with a median follow-up of 55 months, reported comparable findings, indicating a 9% incidence of grade III/IV toxicity [9].

In our study, we compared dysphagia levels at six weeks post-treatment with baseline levels. We found that there was no significant rise in dysphagia when comparing post-treatment scores with those obtained before treatment commenced. There was a notable enhancement in late dysphagia scores compared to dysphagia scores recorded in the final week of radiation therapy for patients. This is believed to reflect the consequential impact of severe acute depletion of mucosal and submucosal stem cells caused by irradiation. Decreasing the dose of the DARS might have facilitated a quicker recovery of mucosal cells, consequently resulting in decreased dysphagia scores. This can be supported by a comparison of the mean dose to the DARS in the 3D-CRT and IMRT groups. In our study, there is a statistically significant difference between the mean dose received by the DARS in the 3D-CRT and IMRT groups (p-value = 0.045).

In our study, no correlation was observed between the utilization of concurrent chemotherapy and dysphagia scores. In a similar study done by Schwartz et al., the authors did not find any difference in dysphagia scores with the addition of concurrent chemotherapy [11]. Forastiere et al. and Eishbruch et al. have both noted a higher occurrence of late dysphagia when concurrent chemotherapy is added. This phenomenon may be attributed to the enhanced impact of radiation on DARSs due to the combined effects of chemotherapy [13,14]. The impact of primary tumors on swallowing dysfunction has been studied by various authors. It was found that the larynx, hypopharynx, base of the tongue, and pharyngeal wall tumors had a higher incidence of swallowing dysfunction compared to other sites. In our study, we did not find any significant correlation between the primary site and dysphagia scores, probably because of the smaller sample size. In our study, there is a significant benefit in the locoregional control with the IMRT group at the end of three months when compared to the 3D-CRT group (p-value = 0.037). Therefore, based on our study, it can be deduced that the dose for DARSs plays a significant role in assessing and understanding swallowing function in head and neck cancers. However, further research is needed to provide additional insights into this matter.

Limitations

The study's limitations include a small sample size and the lack of investigation into the impact of salivary function on dysphagia scores. Although xerostomia could potentially affect dysphagia scores, our study achieved parotid gland dose-sparing goals in most patients, which should mitigate this influence. In addition, the study did not examine the effect of Ryles tube dependency on dysphagia scores, nor did it assess the impact of analgesics. Variability in the primary disease site could also affect DARS sparing, with oral cavity lesions potentially benefiting more than oropharyngeal or hypopharyngeal tumors due to their proximity to DARSs. The study relied on subjective patient-reported questionnaires, without objective assessments, such as video-fluoroscopy or esophagogram, which may limit the findings' accuracy. Lastly, pre-existing conditions affecting the oral phase of swallowing, like trismus or submucous fibrosis, were not accounted for, potentially confounding the assessment of dysphagia.

## Conclusions

Our study concluded that there exists a notable reduction in the mean dose for DARSs within the IMRT group in comparison to the 3D-CRT group. In addition, we observed a significant decrease in the severity of dysphagia among participants in the IMRT group at both three and six months compared to those in the 3D-CRT group. Nonetheless, due to the diverse nature of our study population, we encountered challenges in establishing a definitive correlation between the dose received by DARSs and the severity of dysphagia. Future large-scale studies are warranted to further validate our findings, potentially leading to enhanced preservation of DARSs.
